# Decacarbonyl-1κ^3^ *C*,2κ^3^ *C*,3κ^4^ *C*-μ-hydrido-1:2κ^2^ *H*:*H*-(μ-quinoline-2-thiol­ato-1:2κ^2^ *S*:*S*)diosmium(I)osmium(0)(3 *Os*—*Os*)

**DOI:** 10.1107/S1600536808004881

**Published:** 2008-04-02

**Authors:** Yu Sun, Xiu-Bing Li, Bai-Wang Sun

**Affiliations:** a Ordered Matter Science Research Center, College of Chemistry and Chemical Engineering, Southeast University, Nanjing 210096, People’s Republic of China; b Key Laboratory of Medicinal Chemistry for Natural Resources, Ministry of Education and Department of Chemistry, Yunnan University, Kunming 650091, People’s Republic of China

## Abstract

The title compound, [Os_3_(C_9_H_6_NS)H(CO)_10_], contains a nearly equilateral triangle of Os atoms. Two of the Os atoms are bridged by an S atom of the quinoline-2-thiol­ate ligand. Ten carbonyl groups complete the cluster, resulting in a distorted octa­hedral geometry for each Os atom. The hydride atom, which was located in a difference Fourier map and refined isotropically, bridges the shortest Os–Os edge.

## Related literature

For related literature, see: Begum *et al.* (2007 ▶); Fan *et al.* (2004 ▶); Miyake *et al.* (2007 ▶); Zeller *et al.* (2003 ▶).
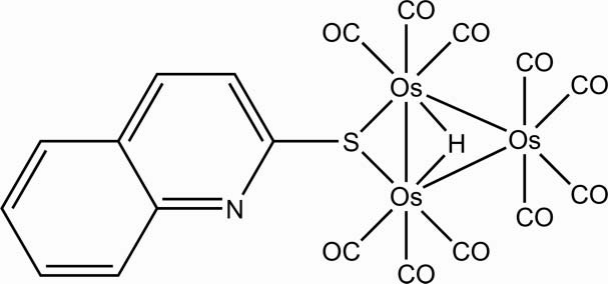


## Experimental

### 

#### Crystal data

[Os_3_(C_9_H_6_NS)H(CO)_10_]
*M* *_r_* = 1012.02
Monoclinic, 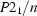

*a* = 9.3593 (5) Å
*b* = 9.4129 (5) Å
*c* = 25.7433 (14) Å
β = 93.045 (1)°
*V* = 2264.7 (2) Å^3^
*Z* = 4
Mo *K*α radiation
μ = 16.94 mm^−1^
*T* = 223 (2) K
0.18 × 0.16 × 0.14 mm

#### Data collection

Rigaku SCXMini 1K CCD area-detector diffractometer
Absorption correction: multi-scan (*CrystalClear*; Rigaku, 2005 ▶) *T*_min_ = 0.058, *T*_max_ = 0.092
13690 measured reflections
4446 independent reflections
4162 reflections with *I* > 2σ(*I*)
*R*_int_ = 0.032

#### Refinement

*R*[*F* ^2^ > 2σ(*F* ^2^)] = 0.024
*wR*(*F* ^2^) = 0.052
*S* = 1.14
4446 reflections
311 parameters
1 restraint
H atoms treated by a mixture of independent and constrained refinement
Δρ_max_ = 0.75 e Å^−3^
Δρ_min_ = −1.24 e Å^−3^

### 

Data collection: *CrystalClear* (Rigaku, 2005 ▶); cell refinement: *CrystalClear*; data reduction: *CrystalClear*; program(s) used to solve structure: *SHELXS97* (Sheldrick, 2008 ▶); program(s) used to refine structure: *SHELXL97* (Sheldrick, 2008 ▶); molecular graphics: *SHELXTL* (Sheldrick, 2008 ▶); software used to prepare material for publication: *SHELXTL*.

## Supplementary Material

Crystal structure: contains datablocks I, global. DOI: 10.1107/S1600536808004881/hy2117sup1.cif

Structure factors: contains datablocks I. DOI: 10.1107/S1600536808004881/hy2117Isup2.hkl

Additional supplementary materials:  crystallographic information; 3D view; checkCIF report

## Figures and Tables

**Table 1:** Selected bond lengths (Å)

Os1—C11	1.892 (6)
Os1—C12	1.900 (6)
Os1—C13	1.922 (6)
Os1—S1	2.4154 (14)
Os1—Os2	2.8399 (3)
Os1—Os3	2.8559 (3)
Os1—H1	1.87 (6)
Os2—C23	1.896 (6)
Os2—C22	1.901 (6)
Os2—C21	1.926 (6)
Os2—S1	2.4144 (13)
Os2—Os3	2.8516 (3)
Os2—H1	1.86 (6)
Os3—C33	1.897 (6)
Os3—C34	1.929 (6)
Os3—C31	1.941 (6)
Os3—C32	1.970 (6)

**Fig. 1.**
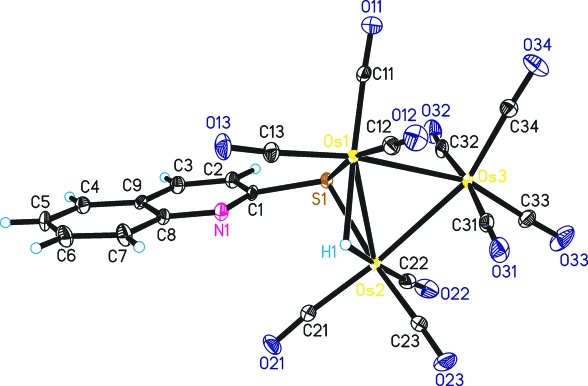
The molecular structure of the title compound. Displacement ellipsoids are drawn at the 30% probability level.

## supplementary crystallographic information

### Comment

In recent years, transition metal–carbonyl clusters have received considerable attention owing to their important role in catalytic reactions (Miyake *et al.*, 2007; Zeller *et al.*, 2003) as well as the preparation of materials with novel magnetic properties (Fan *et al.*, 2004). Different organic ligands containing O and S atoms can stabilize the metal cluster framework by means of chelating and bridging (Begum *et al.*, 2007). We report here the synthesis and structure of the title compound containing a triangle of Os atoms and an organic quinoline-2-thiol ligand.

The S atom of the ligand acts as a bidentate bridge connecting two Os atoms [Os1—S1 = 2.4154 (14) and Os2—S1 = 2.4144 (13) Å]. The molecule of the title compound (Fig. 1) consists of an Os_3_ triangle with ten terminal CO ligands and a substituted quinoline-2-thiol ligand. Each Os atom is in a distorted octahedral geometry, with Os3 bonded to four terminal carbonyl ligands and Os1 and Os2 bonded to three terminal carbonyl ligands and one bridging S atom from the quinoline-2-thiol ligand, respectively. The hydride H atom was crystallographically located and refined and it is found to bridge across the shortest Os1—Os2 edge.

### Experimental

[Os_3_(CO)_10_(MeCN)_2_] (0.120 g, 0.1 mmol) was added to a MeCN solution (10 ml) of quinoline-2-thiol (0.015 g, 0.1 mmol) and the mixture was stirred at room temperature for one hour. Crystals suitable for crystallographic analysis were obtained after two weeks.

### Refinement

H atoms bound to C were positioned geometrically and refined as riding atoms, with C—H = 0.94Å and *U*_iso_(H) = 1.2*U*_eq_(C). The hydride H atom was located from a difference Fourier map and refined isotropically.

### Crystal data

**Table:**

[Os_3_(C_9_H_6_NS)H(CO)_10_]	*F*_000_ = 1808
*M**_r_* = 1012.02	*D*_x_ = 2.968 Mg m^−^^3^
Monoclinic, *P*2_1_/*n*	Mo *K*α radiation λ = 0.71073 Å
Hall symbol: -P 2yn	Cell parameters from 4762 reflections
*a* = 9.3593 (5) Å	θ = 3.0–26.1º
*b* = 9.4129 (5) Å	µ = 16.94 mm^−^^1^
*c* = 25.7433 (14) Å	*T* = 223 (2) K
β = 93.045 (1)º	Block, colourless
*V* = 2264.7 (2) Å^3^	0.18 × 0.16 × 0.14 mm
*Z* = 4	

### Data collection

**Table:**

Rigaku Scxmini 1K CCD area-detector diffractometer	4446 independent reflections
Radiation source: fine-focus sealed tube	4162 reflections with *I* > 2σ(*I*)
Monochromator: graphite	*R*_int_ = 0.032
*T* = 223(2) K	θ_max_ = 26.0º
ω scans	θ_min_ = 2.3º
Absorption correction: multi-scan(CrystalClear; Rigaku, 2005)	*h* = −10→11
*T*_min_ = 0.058, *T*_max_ = 0.092	*k* = −11→8
13690 measured reflections	*l* = −31→31

### Refinement

**Table:**

Refinement on *F*^2^	Secondary atom site location: difference Fourier map
Least-squares matrix: full	Hydrogen site location: inferred from neighbouring sites
*R*[*F*^2^ > 2σ(*F*^2^)] = 0.024	H atoms treated by a mixture of independent and constrained refinement
*wR*(*F*^2^) = 0.052	*w* = 1/[σ^2^(*F*_o_^2^) + (0.0163*P*)^2^ + 2.1547*P*] where *P* = (*F*_o_^2^ + 2*F*_c_^2^)/3
*S* = 1.14	(Δ/σ)_max_ = 0.001
4446 reflections	Δρ_max_ = 0.75 e Å^−^^3^
311 parameters	Δρ_min_ = −1.24 e Å^−^^3^
1 restraint	Extinction correction: none
Primary atom site location: structure-invariant direct methods	

### Fractional atomic coordinates and isotropic or equivalent isotropic displacement parameters (Å^2^)

**Table:**

	*x*	*y*	*z*	*U*_iso_*/*U*_eq_	
Os1	0.76242 (2)	0.01890 (2)	0.169927 (8)	0.01740 (6)	
Os2	0.97667 (2)	−0.15956 (2)	0.130925 (8)	0.01676 (6)	
Os3	1.04641 (2)	0.12428 (2)	0.161547 (8)	0.01774 (6)	
N1	0.5984 (5)	−0.2445 (5)	0.08694 (18)	0.0239 (11)	
S1	0.79273 (14)	−0.03585 (15)	0.07951 (5)	0.0200 (3)	
O11	0.6461 (5)	0.3150 (5)	0.1487 (2)	0.0434 (12)	
O12	0.7862 (5)	0.0836 (5)	0.28569 (17)	0.0401 (11)	
O13	0.4768 (5)	−0.1310 (5)	0.18287 (19)	0.0414 (12)	
O21	0.8805 (5)	−0.4583 (5)	0.09893 (18)	0.0402 (11)	
O22	1.1990 (5)	−0.1242 (5)	0.0512 (2)	0.0465 (13)	
O23	1.1859 (5)	−0.2826 (5)	0.21235 (18)	0.0426 (12)	
O31	1.1025 (5)	−0.0142 (5)	0.26916 (17)	0.0384 (11)	
O32	0.9894 (5)	0.2326 (5)	0.04959 (18)	0.0426 (12)	
O33	1.3613 (5)	0.1450 (6)	0.1407 (2)	0.0605 (17)	
O34	1.0082 (6)	0.4241 (5)	0.20439 (19)	0.0471 (13)	
H1	0.851 (7)	−0.156 (8)	0.184 (3)	0.09 (3)*	
C1	0.6572 (5)	−0.1579 (6)	0.0552 (2)	0.0183 (11)	
C2	0.6192 (6)	−0.1465 (6)	0.0019 (2)	0.0227 (12)	
H2A	0.6674	−0.0837	−0.0195	0.027*	
C3	0.5099 (6)	−0.2299 (7)	−0.0178 (2)	0.0281 (13)	
H3A	0.4821	−0.2252	−0.0534	0.034*	
C4	0.3199 (6)	−0.4058 (7)	−0.0022 (3)	0.0304 (14)	
H4A	0.2871	−0.4038	−0.0374	0.036*	
C5	0.2526 (6)	−0.4889 (7)	0.0322 (3)	0.0349 (16)	
H5A	0.1730	−0.5436	0.0207	0.042*	
C6	0.3013 (7)	−0.4935 (7)	0.0844 (3)	0.0374 (16)	
H6A	0.2530	−0.5506	0.1078	0.045*	
C7	0.4172 (7)	−0.4170 (7)	0.1020 (3)	0.0332 (15)	
H7A	0.4499	−0.4231	0.1371	0.040*	
C8	0.4886 (5)	−0.3279 (6)	0.0673 (2)	0.0197 (12)	
C9	0.4392 (5)	−0.3222 (6)	0.0148 (2)	0.0213 (12)	
C11	0.6869 (6)	0.2026 (7)	0.1567 (2)	0.0267 (13)	
C12	0.7783 (6)	0.0587 (7)	0.2424 (2)	0.0268 (13)	
C13	0.5842 (6)	−0.0799 (7)	0.1754 (2)	0.0279 (13)	
C21	0.9088 (6)	−0.3461 (6)	0.1112 (2)	0.0251 (13)	
C22	1.1165 (6)	−0.1414 (6)	0.0804 (2)	0.0276 (14)	
C23	1.1095 (6)	−0.2351 (6)	0.1818 (2)	0.0245 (13)	
C31	1.0809 (6)	0.0373 (6)	0.2294 (2)	0.0243 (13)	
C32	1.0046 (6)	0.1898 (7)	0.0897 (2)	0.0259 (13)	
C33	1.2440 (6)	0.1335 (7)	0.1489 (3)	0.0311 (15)	
C34	1.0190 (6)	0.3122 (7)	0.1892 (2)	0.0263 (13)	

### Atomic displacement parameters (Å^2^)

**Table:**

	*U*^11^	*U*^22^	*U*^33^	*U*^12^	*U*^13^	*U*^23^
Os1	0.01208 (11)	0.01895 (12)	0.02128 (12)	−0.00154 (8)	0.00173 (8)	−0.00144 (8)
Os2	0.01364 (11)	0.01579 (11)	0.02091 (12)	−0.00091 (8)	0.00137 (8)	−0.00040 (8)
Os3	0.01302 (11)	0.01713 (11)	0.02298 (12)	−0.00306 (8)	0.00002 (8)	−0.00002 (8)
N1	0.021 (2)	0.027 (3)	0.023 (3)	−0.005 (2)	−0.0013 (19)	0.002 (2)
S1	0.0181 (6)	0.0206 (7)	0.0212 (7)	−0.0043 (5)	−0.0009 (5)	0.0022 (5)
O11	0.038 (3)	0.031 (3)	0.061 (3)	0.006 (2)	−0.006 (2)	−0.006 (2)
O12	0.048 (3)	0.050 (3)	0.023 (2)	0.001 (2)	0.005 (2)	−0.007 (2)
O13	0.028 (2)	0.046 (3)	0.051 (3)	−0.017 (2)	0.013 (2)	−0.009 (2)
O21	0.051 (3)	0.023 (2)	0.045 (3)	−0.008 (2)	−0.011 (2)	−0.008 (2)
O22	0.045 (3)	0.037 (3)	0.061 (3)	0.005 (2)	0.036 (3)	−0.001 (2)
O23	0.041 (3)	0.042 (3)	0.044 (3)	0.013 (2)	−0.014 (2)	0.005 (2)
O31	0.046 (3)	0.041 (3)	0.027 (2)	−0.008 (2)	−0.011 (2)	0.002 (2)
O32	0.050 (3)	0.042 (3)	0.035 (3)	−0.017 (2)	−0.004 (2)	0.015 (2)
O33	0.019 (3)	0.075 (4)	0.089 (4)	−0.004 (2)	0.005 (3)	0.034 (3)
O34	0.072 (4)	0.027 (3)	0.043 (3)	0.001 (3)	0.005 (3)	−0.009 (2)
C1	0.012 (2)	0.021 (3)	0.021 (3)	−0.001 (2)	−0.006 (2)	−0.002 (2)
C2	0.023 (3)	0.024 (3)	0.022 (3)	−0.005 (2)	0.000 (2)	0.001 (2)
C3	0.028 (3)	0.032 (3)	0.024 (3)	−0.001 (3)	−0.003 (2)	0.000 (3)
C4	0.026 (3)	0.026 (3)	0.038 (4)	−0.001 (3)	−0.005 (3)	−0.009 (3)
C5	0.017 (3)	0.031 (4)	0.055 (4)	−0.012 (3)	−0.002 (3)	−0.004 (3)
C6	0.035 (4)	0.030 (4)	0.048 (4)	−0.010 (3)	0.012 (3)	0.007 (3)
C7	0.033 (3)	0.035 (4)	0.032 (4)	−0.015 (3)	0.001 (3)	0.006 (3)
C8	0.015 (3)	0.022 (3)	0.023 (3)	−0.003 (2)	0.002 (2)	0.001 (2)
C9	0.013 (3)	0.023 (3)	0.028 (3)	0.001 (2)	0.002 (2)	−0.003 (2)
C11	0.017 (3)	0.030 (4)	0.033 (3)	0.003 (3)	−0.005 (2)	−0.007 (3)
C12	0.022 (3)	0.025 (3)	0.034 (4)	−0.002 (3)	0.009 (3)	−0.003 (3)
C13	0.026 (3)	0.030 (3)	0.028 (3)	−0.004 (3)	0.004 (3)	−0.002 (3)
C21	0.021 (3)	0.027 (3)	0.027 (3)	0.001 (2)	−0.004 (2)	−0.001 (3)
C22	0.023 (3)	0.026 (3)	0.035 (3)	0.005 (3)	0.004 (3)	−0.003 (3)
C23	0.026 (3)	0.018 (3)	0.029 (3)	0.003 (2)	0.000 (3)	−0.003 (2)
C31	0.019 (3)	0.025 (3)	0.028 (3)	−0.007 (2)	−0.001 (2)	0.000 (3)
C32	0.020 (3)	0.029 (3)	0.030 (3)	−0.008 (2)	0.002 (2)	0.002 (3)
C33	0.021 (3)	0.030 (3)	0.041 (4)	0.000 (3)	−0.005 (3)	0.012 (3)
C34	0.026 (3)	0.028 (3)	0.025 (3)	−0.001 (3)	0.001 (2)	−0.001 (3)

### Geometric parameters (Å, °)

**Table:**

Os1—C11	1.892 (6)	O21—C21	1.130 (7)
Os1—C12	1.900 (6)	O22—C22	1.118 (7)
Os1—C13	1.922 (6)	O23—C23	1.127 (7)
Os1—S1	2.4154 (14)	O31—C31	1.140 (7)
Os1—Os2	2.8399 (3)	O32—C32	1.110 (7)
Os1—Os3	2.8559 (3)	O33—C33	1.134 (7)
Os1—H1	1.87 (6)	O34—C34	1.130 (7)
Os2—C23	1.896 (6)	C1—C2	1.404 (8)
Os2—C22	1.901 (6)	C2—C3	1.366 (8)
Os2—C21	1.926 (6)	C2—H2A	0.9400
Os2—S1	2.4144 (13)	C3—C9	1.399 (8)
Os2—Os3	2.8516 (3)	C3—H3A	0.9400
Os2—H1	1.86 (6)	C4—C5	1.361 (9)
Os3—C33	1.897 (6)	C4—C9	1.417 (8)
Os3—C34	1.929 (6)	C4—H4A	0.9400
Os3—C31	1.941 (6)	C5—C6	1.397 (9)
Os3—C32	1.970 (6)	C5—H5A	0.9400
N1—C1	1.297 (7)	C6—C7	1.360 (9)
N1—C8	1.369 (7)	C6—H6A	0.9400
S1—C1	1.799 (5)	C7—C8	1.418 (8)
O11—C11	1.140 (7)	C7—H7A	0.9400
O12—C12	1.138 (7)	C8—C9	1.406 (8)
O13—C13	1.139 (7)		
			
C11—Os1—C12	90.3 (3)	C34—Os3—Os2	158.16 (17)
C11—Os1—C13	97.9 (3)	C31—Os3—Os2	83.02 (17)
C12—Os1—C13	92.6 (2)	C32—Os3—Os2	90.14 (18)
C11—Os1—S1	94.81 (18)	C33—Os3—Os1	161.6 (2)
C12—Os1—S1	168.73 (17)	C34—Os3—Os1	98.51 (17)
C13—Os1—S1	96.58 (18)	C31—Os3—Os1	84.06 (16)
C11—Os1—Os2	137.83 (19)	C32—Os3—Os1	92.31 (16)
C12—Os1—Os2	116.33 (18)	Os2—Os3—Os1	59.679 (7)
C13—Os1—Os2	111.83 (19)	C1—N1—C8	117.6 (5)
S1—Os1—Os2	53.97 (3)	C1—S1—Os2	110.59 (18)
C11—Os1—Os3	90.53 (17)	C1—S1—Os1	110.76 (19)
C12—Os1—Os3	88.91 (17)	Os2—S1—Os1	72.03 (4)
C13—Os1—Os3	171.38 (19)	N1—C1—C2	124.7 (5)
S1—Os1—Os3	81.02 (3)	N1—C1—S1	119.8 (4)
Os2—Os1—Os3	60.085 (8)	C2—C1—S1	115.4 (4)
C11—Os1—H1	176 (3)	C3—C2—C1	117.7 (5)
C12—Os1—H1	88 (2)	C3—C2—H2A	121.1
C13—Os1—H1	86 (2)	C1—C2—H2A	121.1
S1—Os1—H1	86 (2)	C2—C3—C9	120.2 (5)
Os2—Os1—H1	40 (2)	C2—C3—H3A	119.9
Os3—Os1—H1	85 (2)	C9—C3—H3A	119.9
C23—Os2—C22	93.2 (3)	C5—C4—C9	120.1 (6)
C23—Os2—C21	92.0 (2)	C5—C4—H4A	119.9
C22—Os2—C21	97.5 (3)	C9—C4—H4A	119.9
C23—Os2—S1	169.10 (18)	C4—C5—C6	120.4 (6)
C22—Os2—S1	94.50 (18)	C4—C5—H5A	119.8
C21—Os2—S1	94.56 (17)	C6—C5—H5A	119.8
C23—Os2—Os1	115.27 (17)	C7—C6—C5	121.2 (6)
C22—Os2—Os1	135.51 (18)	C7—C6—H6A	119.4
C21—Os2—Os1	113.78 (17)	C5—C6—H6A	119.4
S1—Os2—Os1	54.00 (3)	C6—C7—C8	119.8 (6)
C23—Os2—Os3	91.62 (17)	C6—C7—H7A	120.1
C22—Os2—Os3	87.07 (18)	C8—C7—H7A	120.1
C21—Os2—Os3	173.95 (17)	N1—C8—C9	122.3 (5)
S1—Os2—Os3	81.12 (3)	N1—C8—C7	118.5 (5)
Os1—Os2—Os3	60.236 (8)	C9—C8—C7	119.1 (5)
C23—Os2—H1	85 (2)	C3—C9—C8	117.3 (5)
C22—Os2—H1	172 (3)	C3—C9—C4	123.3 (5)
C21—Os2—H1	90 (2)	C8—C9—C4	119.3 (5)
S1—Os2—H1	86 (2)	O11—C11—Os1	177.7 (5)
Os1—Os2—H1	41 (2)	O12—C12—Os1	179.1 (6)
Os3—Os2—H1	86 (2)	O13—C13—Os1	173.5 (5)
C33—Os3—C34	99.9 (3)	O21—C21—Os2	174.3 (5)
C33—Os3—C31	93.3 (2)	O22—C22—Os2	176.8 (6)
C34—Os3—C31	94.2 (2)	O23—C23—Os2	178.2 (6)
C33—Os3—C32	88.3 (2)	O31—C31—Os3	179.3 (6)
C34—Os3—C32	92.1 (3)	O32—C32—Os3	175.0 (5)
C31—Os3—C32	173.2 (2)	O33—C33—Os3	177.1 (6)
C33—Os3—Os2	101.9 (2)	O34—C34—Os3	177.0 (6)
			
C11—Os1—Os2—C23	128.2 (3)	S1—Os1—Os3—C31	−138.78 (18)
C12—Os1—Os2—C23	4.5 (3)	Os2—Os1—Os3—C31	−85.37 (18)
C13—Os1—Os2—C23	−100.2 (3)	C11—Os1—Os3—C32	−59.4 (3)
S1—Os1—Os2—C23	177.8 (2)	C12—Os1—Os3—C32	−149.7 (3)
Os3—Os1—Os2—C23	76.47 (19)	S1—Os1—Os3—C32	35.40 (19)
C11—Os1—Os2—C22	3.5 (4)	Os2—Os1—Os3—C32	88.80 (18)
C12—Os1—Os2—C22	−120.2 (3)	C11—Os1—Os3—Os2	−148.20 (18)
C13—Os1—Os2—C22	135.1 (3)	C12—Os1—Os3—Os2	121.52 (19)
S1—Os1—Os2—C22	53.1 (3)	S1—Os1—Os3—Os2	−53.41 (3)
Os3—Os1—Os2—C22	−48.2 (3)	C23—Os2—S1—C1	−116.8 (10)
C11—Os1—Os2—C21	−127.3 (3)	C22—Os2—S1—C1	108.1 (3)
C12—Os1—Os2—C21	109.0 (3)	C21—Os2—S1—C1	10.2 (3)
C13—Os1—Os2—C21	4.3 (3)	Os1—Os2—S1—C1	−106.0 (2)
S1—Os1—Os2—C21	−77.71 (19)	Os3—Os2—S1—C1	−165.5 (2)
Os3—Os1—Os2—C21	−179.00 (19)	C23—Os2—S1—Os1	−10.7 (10)
C11—Os1—Os2—S1	−49.6 (3)	C22—Os2—S1—Os1	−145.81 (18)
C12—Os1—Os2—S1	−173.3 (2)	C21—Os2—S1—Os1	116.24 (18)
C13—Os1—Os2—S1	82.0 (2)	Os3—Os2—S1—Os1	−59.50 (2)
Os3—Os1—Os2—S1	−101.29 (4)	C11—Os1—S1—C1	−105.0 (3)
C11—Os1—Os2—Os3	51.7 (2)	C12—Os1—S1—C1	138.3 (10)
C12—Os1—Os2—Os3	−72.0 (2)	C13—Os1—S1—C1	−6.5 (3)
C13—Os1—Os2—Os3	−176.69 (19)	Os2—Os1—S1—C1	105.81 (19)
S1—Os1—Os2—Os3	101.29 (4)	Os3—Os1—S1—C1	165.19 (19)
C23—Os2—Os3—C33	60.7 (3)	C11—Os1—S1—Os2	149.15 (17)
C22—Os2—Os3—C33	−32.5 (3)	C12—Os1—S1—Os2	32.5 (10)
S1—Os2—Os3—C33	−127.50 (19)	C13—Os1—S1—Os2	−112.27 (19)
Os1—Os2—Os3—C33	179.09 (19)	Os3—Os1—S1—Os2	59.38 (2)
C23—Os2—Os3—C34	−115.1 (5)	C8—N1—C1—C2	−2.0 (8)
C22—Os2—Os3—C34	151.7 (5)	C8—N1—C1—S1	175.3 (4)
S1—Os2—Os3—C34	56.7 (5)	Os2—S1—C1—N1	51.1 (5)
Os1—Os2—Os3—C34	3.3 (5)	Os1—S1—C1—N1	−26.7 (5)
C23—Os2—Os3—C31	−31.2 (2)	Os2—S1—C1—C2	−131.3 (4)
C22—Os2—Os3—C31	−124.4 (2)	Os1—S1—C1—C2	150.8 (4)
S1—Os2—Os3—C31	140.59 (17)	N1—C1—C2—C3	1.9 (9)
Os1—Os2—Os3—C31	87.18 (17)	S1—C1—C2—C3	−175.6 (4)
C23—Os2—Os3—C32	149.0 (2)	C1—C2—C3—C9	0.3 (9)
C22—Os2—Os3—C32	55.8 (2)	C9—C4—C5—C6	−0.5 (10)
S1—Os2—Os3—C32	−39.19 (17)	C4—C5—C6—C7	−0.8 (11)
Os1—Os2—Os3—C32	−92.60 (17)	C5—C6—C7—C8	1.6 (10)
C23—Os2—Os3—Os1	−118.41 (18)	C1—N1—C8—C9	0.1 (8)
C22—Os2—Os3—Os1	148.44 (18)	C1—N1—C8—C7	−176.2 (6)
S1—Os2—Os3—Os1	53.42 (3)	C6—C7—C8—N1	175.4 (6)
C11—Os1—Os3—C33	−151.0 (6)	C6—C7—C8—C9	−1.0 (9)
C12—Os1—Os3—C33	118.7 (6)	C2—C3—C9—C8	−2.0 (8)
S1—Os1—Os3—C33	−56.2 (6)	C2—C3—C9—C4	176.3 (6)
Os2—Os1—Os3—C33	−2.8 (6)	N1—C8—C9—C3	1.9 (8)
C11—Os1—Os3—C34	33.0 (3)	C7—C8—C9—C3	178.2 (6)
C12—Os1—Os3—C34	−57.3 (3)	N1—C8—C9—C4	−176.5 (5)
S1—Os1—Os3—C34	127.82 (18)	C7—C8—C9—C4	−0.2 (8)
Os2—Os1—Os3—C34	−178.77 (17)	C5—C4—C9—C3	−177.3 (6)
C11—Os1—Os3—C31	126.4 (3)	C5—C4—C9—C8	1.0 (9)
C12—Os1—Os3—C31	36.1 (3)		

## References

[bb1] Begum, N., Das, U. K., Hassan, M., Hogarth, G., Kabir, S. E., Nordlander, E., Rahman, M. A. & Tocher, D. A. (2007). *Organometallics*, **26**, 6462–6472.

[bb2] Fan, W., Zhang, R., Leong, W. K. & Yan, Y. K. (2004). *Inorg. Chim. Acta*, **357**, 2441–2450.

[bb3] Miyake, Y., Nomaguchi, Y., Yuki, M. & Nishibayashi, Y. (2007). *Organo­metallics*, **26**, 3611–3613.

[bb4] Rigaku (2005). *CrystalClear* Rigaku Corporation, Tokyo, Japan.

[bb5] Sheldrick, G. M. (2008). *Acta Cryst.* A**64**, 112–122. 10.1107/S0108767307043930 18156677

[bb6] Zeller, M., Hunter, A. D., Regula, J. L. & Szalay, P. S. (2003). *Acta Cryst.* E**59**, m975–m976.

